# Disposable E-Cigarettes and Associated Health Risks: An Experimental Study

**DOI:** 10.3390/ijerph191710633

**Published:** 2022-08-26

**Authors:** Hsien-Chang Lin, Anne Buu, Wei-Chung Su

**Affiliations:** 1 Department of Applied Health Science, School of Public Health, Indiana University Bloomington, 1025 E. 7th St., SPH 116, Bloomington, IN 47405, USA; 2 Department of Health Promotion and Behavioral Sciences, University of Texas Health Science Center, 7000 Fannin St., Houston, TX 77030, USA; 3 Department of Epidemiology, Human Genetics & Environmental Sciences, University of Texas Health Science Center, 1200 Pressler St., Houston, TX 77030, USA

**Keywords:** disposable e-cigarettes, aerosol toxicity, health risk, particle size distribution, respiratory deposition

## Abstract

The use of electronic nicotine delivery systems (ENDS), including disposable e-cigarettes, has been prevalent. Existing chemical analyses of ENDS focused on e-liquids rather than aerosols and failed to consider particle sizes and aerosol respiratory deposition fractions, which are key factors for inhalation doses. This study investigated the organic chemical and metal constituents in size-segregated ENDS aerosol and assessed the deposited doses and health risks of these substances. Aerosol chemical analyses were conducted on two popular disposable ENDS products: Puff Bar (Grape) and Air Bar (Watermelon Ice). An ENDS aerosol was generated and delivered into a Micro-Orifice Uniform Deposit Impactor to collect size-segregated aerosol samples, in which organic chemicals and metals were analyzed. Daily and lifetime doses for each chemical were estimated. Cancer and non-cancer risk assessments were conducted based on the deposited doses. We found that e-cigarette aerosol contains certain harmful organic chemicals and metals documented to result in respiratory problems. Estimated respiratory cancer risks corresponding to chromium from both ENDS products and nickel from Air Bar (Watermelon Ice) were substantially above the conventionally acceptable risk. The method, findings, and implications can contribute to the extant literature of ENDS toxicity studies as well as inform tobacco regulation and future large-scale studies.

## 1. Introduction

Electronic nicotine delivery systems (ENDS) use, including the use of e-cigarettes, is prevalent among youth and young adults and has become a public health issue. According to our secondary analysis of the 2019 National Health Interview Survey data, the prevalence rates of ever use and current use of ENDS among young adults were 32.4% and 9.4%, respectively, following an increasing trend over the past decade [1]. Further, a study using data from the 2019 National Youth Tobacco Survey revealed that 27.5% of high school students reported current e-cigarette use; among them, 34.2% reported frequent e-cigarette use (i.e., used on 20 or more days in the past 30 days) [2]. ENDS products are particularly appealing to youth and young adults due to their customizable and novel characteristics [3,4,5], lower perceived risk [6,7], and higher social acceptability than combustible cigarettes [8].

ENDS products are diverse and have been rapidly evolving from cigarette-like disposable products (first generation) to cartridge systems (second generation), to tank systems (third generation), and to nicotine salt devices such as JUUL (fourth generation). Recently, the popularity of JUUL has been caught up by JUUL-like disposable e-cigarette devices (hereafter, “disposable ENDS”) due to their flavoring options, eye-catching design and packaging, low price, and convenience (i.e., all-in-one, self-contained, no filling of e-liquid and no battery charging) [2,9]. The tobacco industry has shown remarkable ingenuity in providing new/modified products following regulations [10]. For example, as of when this study was conducted in early 2022, disposable ENDS products are not restricted by the federal flavor regulation due to the FDA’s narrow definition of a cartridge [11]. In fact, 85.8% of current youth users purchase disposable ENDS with fruit flavors [12].

Studies have found that ENDS products contain flavoring chemicals, of which some are associated with respiratory irritation or harm, such as δ-dodecalactone, menthol, benzyl alcohol, and corylone [13,14,15,16]. Furthermore, the heating elements (e.g., atomizer/coil) and tanks of ENDS devices consist of metals that could be released into e-liquid and aerosol during use, with chromium and nickel being the leading contributors to cancer risk [17,18]. However, existing chemical analyses conducted for ENDS products, including disposable ENDS, mainly focused on *e-liquid* that does not necessarily reflect the chemicals in ENDS *aerosol* that actually deposit in ENDS users’ airways [15,19]. Although there are a handful of studies that analyzed ENDS aerosol [20,21,22] and assessed the associated health risks [18], these studies failed to consider the particle size of ENDS aerosol and the size-related respiratory deposition fractions while estimating the health risks. This is a serious issue as the size of the particles directly affects the respiratory deposition location and deposition fraction in the airways. In fact, the ENDS aerosol generated from vaping is a mix of liquid droplets with the aerosol particle diameter ranging from ultrafine (less than 0.1 µm) to micron sizes (up to around 4 µm). Thus, inhaled ENDS aerosol is expected to enter and deposit in different human airway regions with various deposited doses. Importantly, it is expected that most of the ENDS aerosol will deposit in the lower respiratory tract, likely making negative impacts on health because of the enormous airway surface area in the alveolar region [23]. The deposited dose is the antecedent of the internal dose prior to the consideration of respiratory clearance and absorption rate and hence may serve as the pivot index of vaping-associated health risk assessment. The ENDS aerosol size distribution and respiratory deposition fractions are the key factors to accurately estimating this pivot index [23], and thus it should be included in health risk assessment.

This study filled the knowledge gaps by using an improved approach to estimate the deposited doses of chemicals in two commonly adopted disposable ENDS products and their associated cancer and non-cancer health risks. The experimental approach considered the size-dependent aerosol respiratory deposition fractions and conducted a size-segregated chemical constituent analysis on ENDS aerosol. Findings from our study could improve the accuracy of estimating doses of ENDS chemicals deposited in the vaper’s lungs, which in turn could inform tobacco regulations and future large-scale studies that assess health risks associated with disposable ENDS product use.

## 2. Materials and Methods

### 2.1. Size-Segregated ENDS Aerosol Sample Collection

Aerosol chemical analyses in this study were conducted on the following two popular disposable ENDS products: Puff Bar (Grape) and Air Bar (Watermelon Ice). The selection of these two disposable ENDS products was based on the prevalence of disposable ENDS products from a separate study by the research team that examined college students’ ENDS use behaviors. These two disposable ENDS products were the most commonly adopted ones among the study participants.

In this study, size-segregated ENDS aerosol samples were collected by using a Micro-Orifice Uniform Deposit Impactor (MOUDI 110-R, MSP Co., Shoreview, MN, USA). The MOUDI is capable of collecting aerosol samples with particle diameters ranging from 0.056 to 18 µm in its 11 collection stages (i.e., size-segregated sampling). It outperforms common direct-reading aerosol instruments such as particle counters and spectrometers that cannot handle freshly generated e-cigarette aerosol with an extremely high aerosol concentration. Each MOUDI stage has a collectible particle size range, and an average lung deposition fraction can be calculated based on that particle size range (see Appendix A Table A1). ENDS aerosol samples collected by MOUDI in different stages were used for particle size distribution calculations and chemical composition analyses.

To collect disposable ENDS aerosol samples, polytetrafluoroethylene (PTFE) membrane filters (PALL Co., Port Washington, NY, USA) were placed on each MOUDI stage, and the MOUDI was operated at the 30 L/min sampling flow rate. A 100 mL syringe was used to draw ENDS aerosol from the two disposable ENDS products and then pump the ENDS aerosol directly into MOUDI for sample collection. This procedure was repeated 10 times (10 puffs) to accumulate a sufficient amount of ENDS aerosol on each MOUDI stage for subsequent weight and substance analysis. After the sample collection experiment, PTFE filters with collected ENDS aerosol samples were unloaded from the MOUDI, individually weighted by a microbalance, and then sent to labs for organic chemical analysis and metal analysis to identify size-dependent chemical constituents in ENDS aerosol. The sample collection was conducted three times for each ENDS product to ensure data reliability and replicability. The ENDS aerosol particle size distribution and the size-dependent chemical constituent data play a key role in estimating the deposited dose of a specific ENDS chemical in the vaper’s lungs.

### 2.2. Organic Chemical and Metal Analysis

The organic chemical analysis for ENDS aerosol was conducted using gas chromatography-mass spectrometry (GC/MS) to detect the mass of organic chemicals such as nicotine, PG, VG, and flavoring agents on MOUDI filters for acquiring size-dependent information. Specifically, the two disposable ENDS were disassembled to locate the e-liquid-soaked vaping wicks. The major chemical components in the e-liquid were first extracted with acetonitrile and analyzed by GC/MS. Then, the ENDS aerosol samples on MOUDI filters were extracted in the same manner and concentrated for GC/MS analysis. The GC/MS chemical analysis was equipped with an electron ionization ion source (GC/EI-MS, Agilent 6890N GC, and 5975 inert XL MSD), and chemicals were all quantified using authentic or surrogate standards. Detailed procedures for organic analysis are described elsewhere [23]. In the organic chemical analysis, the recovery of target analytes was determined by spiking a known amount of chemicals onto blank filters (*n* = 3) and extracting them with the same procedures as collecting aerosol samples. The metal analysis was conducted using Inductively Coupled Plasma Mass Spectrometry (ICP/MS) to detect the mass of hazardous metals such as nickel, chromium, and lead on different MOUDI filters for obtaining size-dependent information. The MOUDI filters were first processed for acid leach and digestion using ultra-clean double distilled HNO_3_ and HF, and then the digested sample solutions were evaporated to incipient dryness. A previously well-established protocol was followed for the ICP/MS analysis using the ICP/MS Triple Quad (Model 8800, Agilent Technologies, Santa Clara, CA, USA) [24]. In this way, metal elements down to sub-ppb (ng/g) levels with ±5% of precision can be acquired.

The focus of the lab analysis in this study is the harmful organic chemicals and metals previously identified in ENDS aerosol and published in the literature [14,18,25,26,27,28,29]. Therefore, the design of our organic chemical analysis was focused on nicotine, carrier solvent (e.g., glycerol, propylene glycol, formaldehyde, acetaldehyde, acrolein, acetone, methylglyoxal, benzoic acid, and triethyl citrate), as well as flavoring and other chemicals (e.g., ethylvanillin, vanillin, cinnamaldehyde, menthol, acetoin, citral, benzyl alcohol, benzaldehyde, diacetyl, ethyl acetate, ethyl maltol, triacetin, methyl anthranilate, methyl dihydrojasmonate, melonal, corylone, δ-dodecalactone, α-terpineol, γ-decalactone, and 3-hexen-1-ol). By the same token, more than 20 metals were detected in our metal analysis, but the focus of this study was on nickel (Ni), manganese (Mn), zinc (Zn), chromium (Cr), and lead (Pb).

### 2.3. Estimating Deposited Doses of ENDS Substances

To estimate the health risks associated with the inhalation of ENDS substances in vapers’ lungs, the average daily dose (ADD) and lifetime average daily dose (LADD) were calculated. ADD and LADD were calculated based on the following conventional equations:

(1) $$ADD=\frac{C\times CR\times CT}{BW\times AT}$$

(2) $$LADD=\frac{C\times CR\times CT}{BW\times LE}$$

where C is the exposure concentration (ng/m^3^), CR is the contact rate (m^3^/day), CT is the contact time, which is assumed to be 30 years (10,950 days) for the high-end vaper population, BW is the body weight (70 kg), AT is the average time, which equals to CT (10,950 days), and LE is the lifetime expectancy, which is 70 years (25,550 days) according to the USEPA default exposure assumptions. In the case of vaping, the term C×CR is equal to the daily intake of the ENDS substance (ng/day), and the daily intake of the substance through vaping should come from the total puffs of vaping in one day. Based on the literature, the reported average daily puffs of a vaper is around 163 puffs/day [30]. Therefore, based on the experimental design and the results of this study, Equations (1) and (2) can then be modified for the vaping setting as follows:

(3) $$ADD_{j}=\underset{i}{\sum }ADD_{i,j}=\frac{0.1M_{i,j}\times CR_{p}\times CT}{BW\times AT}$$

(4) $$LADD_{j}=\underset{i}{\sum }LADD_{i,j}=\frac{0.1M_{i,j}\times CR_{p}\times CT}{BW\times LE}$$

where $M_{i,j}$ is the mass of a specific ENDS substance *j* (organic chemical or metal) in the ENDS aerosol found on the MOUDI stage *I* (*i* = 1 to 11) from the GC/MS and ICP/MS analyses. Since $M_{i,j}$ is based on 10 puffs, 0.1$M_{i,j}$ is equal to the mass of the ENDS substance in one puff. $CR_{p}$ is the ENDS aerosol contact rate in the unit of puff/day. Based on the literature, $CR_{p}$ was set to be 163 puffs/day [30]. Based on these, $ADD_{i,j}$ reflects the average daily dose of a specific substance *j* contained in ENDS aerosol within a size range *i* (MOUDI stage *i*); $LADD_{i,j}$ is the lifetime average daily dose of a specific substance *j* contained in ENDS aerosol within a size range *i*. The summation in Equations (3) and (4) indicates that the dose of a specific ENDS substance in an active vaper’s lungs is calculated by aggregating the doses from all 11 individual MOUDI stages ($\sum ADD_{i,j}$ and $\sum LADD_{i,j}$).

Equations (3) and (4) can be further modified by considering the important factor of aerosol respiratory deposition fractions to estimate the deposited dose in the vaper’s lungs (i.e., the dose contributed by ENDS aerosol that is actually deposited in the airways) as follows:

(5) $$AD{D^{\prime }}_{j}=\underset{i}{\sum }ADD_{i,j}\times DF_{i}$$

(6) $$LAD{D^{\prime }}_{j}=\underset{i}{\sum }LADD_{i,j}\times DF_{i}$$

where $DF_{i}$ is the mean respiratory deposition fraction (from 0.0 to 1.0) for the collectible aerosol size of MOUDI stage *i*. The human respiratory system used in this study covers the tracheobronchial airways to the alveolar region, and the corresponding published data of aerosol respiratory deposition fractions were adopted for acquiring the mean deposition fractions (see Appendix A Table A1 for the estimated $DF_{i}$) [23]. In this way, $AD{D^{\prime }}_{j}$ and $LAD{D^{\prime }}_{j}$ take into account the realistic size-dependent mass fractions of substance in ENDS aerosol as well as the fraction of the inhaled ENDS aerosol that indeed contributes to the deposited dose.

### 2.4. Estimating Cancer and Non-Cancer Risks

The estimated $AD{D^{\prime }}_{j}$ from Equation (5) was then used to calculate the hazard quotient (HQ) for assessing the non-cancer health risk induced by the substance *j* in a disposable ENDS product (organic chemical or metal) as follows:

(7) $$HQ_{j}=\frac{AD{D^{\prime }}_{j}}{RfD_{j}}\;\;\;\;\;\;\;\;\;\;\{\begin{array}{c} <1\;\;\mathrm{No}\;\mathrm{adverse}\;\mathrm{health}\;\mathrm{effect}\;\mathrm{is}\;\mathrm{expected}\\ >1\;\;\mathrm{Adverse}\;\mathrm{health}\;\mathrm{effect}\;\mathrm{is}\;\mathrm{potential}\;\;\;\;\;\; \end{array}$$

where $RfD_{j}$ is the reference dose (RfD) published for the ENDS substance *j*. Note that in the case that only the reference concentration (RfC) was available for a particular substance, the RfC was converted to RfD using a reasonable inhalation rate of 20 m^3^/day and a default body weight of 70 kg (i.e., *RfD* = *RfC* × 20/70) [18]. Further, the hazard index (HI) was applied to the substances, which induce similar non-cancer health effects on the same target organ/system (e.g., respiratory system). Therefore, the HI is the summation of all related $HQ_{j}$ ($HI=\sum HQ_{j}$). When the HQ or HI exceeds 1.0, it indicates that adverse non-cancer health effects are potential for users of disposable ENDS.

$LAD{D^{\prime }}_{j}$ that was calculated in Equation (6) was used to assess the risk of respiratory cancers associated with disposable ENDS product use as follows:

(8) $$Cancer\;Risk_{j}=LAD{D^{\prime }}_{j}\times CSF_{j}\;\;\;\;\;\;\;\;\;\{\begin{array}{c} <10^{-6}\;\;\mathrm{Acceptable}\;\mathrm{cancer}\;\mathrm{risk}\;\;\;\;\;\\ >10^{-6}\;\;\mathrm{Unacceptable}\;\mathrm{cancer}\;\mathrm{risk} \end{array}$$

where $CSF_{j}$ is the published cancer slope factor (i.e., cancer potency) of a specific ENDS substance *j* (organic chemical or metal). For cancer risk assessment, the estimated cancer risk that exceeds 10^−6^ (one in one million) indicates an unacceptable cancer risk for users of disposable ENDS. The values of RfD, RfC, and CSF in Equations (7) and (8) for cancer and non-cancer risk assessments were compiled from US EPA and CalEPA official websites [31,32,33,34].

## 3. Results

### 3.1. Detected Substances in ENDS Aerosol

Table 1 and Table 2 show the (selected) organic chemicals and metals detected from the aerosol generated from the two disposable ENDS products, Puff Bar (Grape) and Air Bar (Watermelon Ice), respectively. The quantified results are expressed as the mass of the substance detected on the filter (ng), which was the average of triplicate filter samples of the same MOUDI stage. For organic compounds, based on our chemical analysis, both Puff Bar (Grape) and Air Bar (Watermelon Ice) contain nicotine, propylene glycol (PG), glycerol (VG), benzoic acid, triethyl citrate, ethyl maltol, and 3-hexen-1-ol. It is known that PG and VG are carrier solvents commonly used in the e-liquid of ENDS products. In addition, Puff Bar also contains methyl anthranilate, α-terpineol, and perillartine, whereas Air Bar also contains benzyl alcohol, vanillin, melonal, methyl dihydrojasmonate, and γ-decalactone. Organic chemicals such as vanillin, melonal, ethyl maltol, and benzyl alcohol are added as flavoring agents [35]. For metal analysis, we only listed metals that have been documented to be associated with adverse health effects in Table 1 and Table 2. Metals detected on filters include chromium, nickel, manganese, lead, aluminum, and zinc, all of which have been documented to negatively impact the respiratory system (and even lung cancer) and the central nervous system [18]. Table 1 and Table 2 also present the mass distribution of ENDS aerosol collected by MOUDI based on the collectible aerosol size ranges. Overall, the mass of organic chemicals was normally distributed and peaked around stage 6 (collectible aerosol size of 1.8 ≥ *d* ≥ 1.0 µm), which is proportional to the mass distribution (equal to particle size distribution) of the ENDS aerosol. However, we did not observe the metals following such a distribution. With the above size-dependent substance constituent data, the dose of a specific ENDS-related substance (organic chemical or metal) deposited in the vaper’s lungs as well as the associated health risks can then be estimated.

### 3.2. Estimated Respiratory Deposited Doses of ENDS Substances

Table 3 and Table 4 show the estimated ADD and LADD for those organic chemicals and metals listed in Table 1 and Table 2. The estimated ADD and LADD were calculated based on Equations (1)–(6), taking account of realistic ENDS usage parameters, aerosol size-dependent substance composition, and the conventional aerosol respiratory deposition fractions. The ADD and LADD measure the respiratory deposited dose of the ENDS substance of interest. As shown in Table 3 and Table 4, the deposited doses corresponding to nicotine, propylene glycol, glycerol, and benzoic acid were relatively higher than those of other organic chemicals. Among the metals, zinc, aluminum, nickel, and chromium tended to have relatively higher deposited doses. In general, Air Bar (Watermelon Ice) resulted in higher deposited doses of both organic chemicals and metals compared to Puff Bar (Grape). Particularly, ENDS aerosol generated from Air Bar (Watermelon Ice) contained higher estimated doses of nicotine, propylene glycol, glycerol, chromium, nickel, manganese, lead, aluminum, and zinc, compared to that from Puff Bar (Grape).

### 3.3. Estimated Cancer and Non-Cancer Risks of ENDS Substances

The cancer and non-cancer risks for the selected metals found in ENDS aerosol were estimated based on the corresponding ADD and LADD and the results are presented in Table 3 and Table 4. These tables also show the values of CSF that were published by the US EPA and CalEPA and were applied in our cancer risk estimation [32]. Among the metals found in the aerosol generated by Air Bar (Watermelon Ice), the estimated cancer risks of chromium and nickel were 1.0 × 10^−3^ and 1.5 × 10^−6^, respectively, which are considered substantially above the acceptable risk of 10^−6^. For the metals in the aerosol generated by Puff Bar (Grape), the estimated respiratory cancer risk corresponding to chromium was 3.9 × 10^−4^, which also exceeds the conventional acceptable risk. For the non-cancer risk estimation (measured by HQ), the RfD (or RfC) corresponding to those metals published by the US EPA and CalEPA were used (listed in Table 3 and Table 4) [31,33,34]. The results of the non-cancer risk assessment show that the values of HQ for all the metals in Table 3 and Table 4 were all less than 1.0, indicating that none of the metals alone would induce a significant impact on the respiratory system or CNS. However, when summing up the non-cancer health risks corresponding to the same target organ, it was found that chromium and nickel in the aerosol generated by Air Bar (Watermelon Ice) posed a respiratory risk of HI = 1.14, which is above the acceptable level of 1.0, indicating a potential risk for adverse respiratory effects (non-cancer) among people who regularly use Air Bar (Watermelon Ice). Similar adverse effects were, however, not found in the aerosol generated by Puff Bar (Grape).

## 4. Discussion

The results obtained from this experimental study filled the literature gaps by providing a more accurate estimation of the respiratory deposited dose and associated cancer and non-cancer health risks related to disposable ENDS use. By acquiring size-segregated chemical constituent data from ENDS aerosol and considering aerosol size-dependent respiratory deposition fraction, the health risks of disposable ENDS products can be accurately assessed. A major strength of this approach is that it takes into account the amount of ENDS substances that actually deposit in human airways.

Our chemical analysis findings verified that e-cigarette aerosol contains harmful organic chemicals and metals (e.g., benzyl alcohol, γ-decalactone, chromium, nickel) that are documented to cause respiratory problems and even lung cancer. We also found manganese and lead to appear in ENDS aerosol, which has known negative effects on human CNS. Among the detected metals, we found that the estimated respiratory cancer risks corresponding to chromium from both disposable ENDS products were substantially above the acceptable risk. The respiratory cancer risk corresponding to nickel in the aerosol from Air Bar (Watermelon Ice) was also substantially above the acceptable risk. Furthermore, neither chromium nor nickel alone in the two disposable ENDs products was associated with unacceptable non-cancer health risks. Yet, when summing up their individual non-cancer risks with the same target organ, we found chromium and nickel in the ENDS aerosol generated by Air Bar (Watermelon Ice) to have an additive effect on the respiratory system. These results echoed the growing evidence supporting the notion that carcinogenic and harmful chemicals are indeed concerns for regular ENDS users [18,25,35]. Notably, our estimated cancer and non-cancer risks were generally lower than those from Fowles et al.’s study [18], possibly due to different types of ENDS and methods to obtain metal concentrations. Our estimations are likely to be more accurate though because they were based on the estimated doses of chemicals deposited in the human airways as well as the composition of ENDS substances experimentally analyzed and found in the ENDS aerosol.

A unique contribution of this study is that the estimation of deposited doses and associated health risks was factored in the particle size of the aerosol. The method used in this study could be further applied to investigating aerosol toxicity for other ENDS products. Specifically, the size-dependent constituent data obtained from our chemical analysis provided useful information to correctly estimate the dose of specific ENDS chemicals in the human airway region. Based on the data obtained from the MOUDI size-segregate sample collection, we found that the particle size distributions of almost all organic chemicals are proportional to the ENDS aerosol mass distribution. The organic chemicals contained in ENDS aerosol were normally distributed and generally peaked around MOUDI Stage 6 (collectible aerosol size: from 1.0 to 1.8 µm). Given the information on size-dependent aerosol respiratory deposition (reported in Appendix A Table A1), this implies that 16% of the most abundant ENDS aerosol inhaled (1.0–1.8 µm) would deposit in the vaper’s lungs within the tracheobronchial airways and alveolar region.

In addition to the improved experimental method, this study has important policy implications. This study found that harmful and potentially harmful organic compounds and metals were detected in the aerosol from the two disposable ENDS, with a few metals yielding over-acceptable cancer and/or non-cancer risks. These results stress the importance of regulating such harmful and potentially harmful chemicals. In addition to verifying the findings of previous studies that heating elements and flavoring agents in e-liquids of ENDS products are sources of some harmful chemicals [18,25], our study further developed a methodology to more accurately estimate doses of ENDS-related substances that could better inform tobacco product regulation by providing a more accurate estimation of toxicant levels. Furthermore, as the FDA has banned flavored ENDS products except for disposable ENDS due to the FDA’s narrow definition of an e-cigarette cartridge [11], findings from our study that focused specifically on disposable ENDS provided an additional scientific foundation for the FDA to consider extending the flavor ban to disposable ENDS products. In addition, because ENDS companies commonly advertise that ENDS contain nicotine, flavoring chemicals, and carrier solutions (e.g., propylene glycol) but downplay the existence of harmful substances in ENDS aerosol [36], stricter product labeling requirements and effective counter communication campaigns are imperative.

This study has limitations that need to be acknowledged. First, although we investigated the aerosol from two commonly adopted disposable ENDS, they did not represent all ENDS products that vary by flavors, models, and brands. Different product characteristics may be associated with different health risks. For example, one study that examined benzaldehyde in ENDS-generated aerosols found that the highest levels of benzaldehyde were detected in cherry-flavored ENDS products [37]. Future studies are needed to systematically investigate aerosol toxicity coupled with product characteristics (e.g., flavor, model, power voltage) to inform additional tobacco regulatory actions. Second, our assessment of cancer and non-cancer health risks associated with organic compounds and metals from ENDS aerosol were based on documented cancer potency (CSP) and RfD (or RfC) information provided by EPA and CalEPA. However, such information has not been established for most of the organic chemicals detected in this study. Thus, although daily doses such as ADD and LADD can be estimated for all organic chemicals found in ENDS aerosol, associated health risk assessments can only be completed for some chemicals. Future studies are needed to further investigate health risks associated with other chemicals when their toxicity information (i.e., CSP, RfD, and RfC) becomes available. Finally, using a syringe to draw aerosol from the ENDS products to generate ENDS aerosol might not represent the real situation of vaping. However, this method is a simple and useful way to efficiently generate an aerosol from ENDS products. For future studies, commercially available e-cigarette puffing machines such as CSM-eSTEP (CH Technology USA, Inc., Northeastern, New Jersey) can be acquired to generate a more representative and reliable ENDS aerosol for related experiments.

## 5. Conclusions

This study provides valuable experimental results on the respiratory deposited dose associated with disposable ENDS product use with consideration of the aerosol size-dependent ENDS substance constituents and aerosol respiratory deposition fractions, which yielded more accurate cancer and non-cancer risk estimations. The method and findings from the study can contribute to the extant literature on ENDS aerosol toxicity and can inform future large-scale studies that investigate health risks associated with ENDS. The findings can also inform tobacco regulatory efforts such as extending the federal flavor ban to disposable ENDS products, implementing stricter product labeling requirements, and developing effective communication campaigns to combat the public health impact of these novel ENDS products.

## Figures and Tables

**Table 1:** Substances detected in size-segregated ENDS aerosol samples generated by Air Bar (Watermelon Ice).

	Mass (ng) Based on 10 Puffs										
MOUDI Stage	1	2	3	4	5	6	7	8	9	10	11
Collectible Size Range (µm)	*d* ≥ 18	18 ≥ *d* ≥ 10	10 ≥ *d* ≥ 5.6	5.6 ≥ *d* ≥ 3.2	3.2 ≥ *d* ≥ 1.8	1.8 ≥ *d* ≥ 1.0	1.0 ≥ *d* ≥ 0.56	0.56 ≥ *d* ≥ 0.32	0.32 ≥ *d* ≥ 0.18	0.18 ≥ *d* ≥ 0.1	0.1 ≥ *d* ≥ 0.0056
Sample Mass	3.48 × 10^3^	5.13 × 10^3^	1.65 × 10^4^	1.13 × 10^5^	5.79 × 10^5^	1.02 × 10^6^	6.33 × 10^5^	2.81 × 10^5^	6.38 × 10^4^	1.04 × 10^4^	3.66 × 10^3^
Nicotine	4.86 × 10^2^	6.99 × 10^2^	1.36 × 10^3^	7.61 × 10^3^	2.64 × 10^4^	4.88 × 10^4^	2.76 × 10^4^	1.45 × 10^4^	2.98 × 10^3^	4.27 × 10^2^	2.92 × 10^2^
Propylene glycol (PG)	3.16 × 10^1^	6.23 × 10^1^	3.70 × 10^2^	9.19 × 10^3^	4.00 × 10^4^	6.93 × 10^4^	4.65 × 10^4^	2.34 × 10^4^	6.70 × 10^3^	1.69 × 10^2^	9.99 × 10^1^
Glycerol (VG)	2.34 × 10^2^	1.82 × 10^3^	8.29 × 10^3^	9.11 × 10^4^	4.83 × 10^5^	8.43 × 10^5^	5.26 × 10^5^	2.24 × 10^5^	4.68 × 10^4^	3.69 × 10^2^	3.69 × 10^2^
Benzoic acid	BDL	9.62 × 10^1^	7.18 × 10^2^	3.48 × 10^3^	2.37 × 10^4^	5.05 × 10^4^	2.30 × 10^4^	1.23 × 10^4^	1.06 × 10^3^	1.06 × 10^2^	8.82 × 10^1^
Triethyl citrate	BDL	BDL	1.89	BDL	1.19 × 10^1^	3.02 × 10^1^	1.80 × 10^1^	2.30 × 10^1^	1.95 × 10^1^	1.41 × 10^1^	2.64 × 10^1^
Ethyl maltol	BDL	BDL	4.48 × 10^−1^	8.75	1.54 × 10^2^	1.41 × 10^2^	1.82 × 10^2^	7.05 × 10^1^	2.94 × 10^1^	1.60	2.23 × 10^−1^
Benzyl alcohol	BDL	BDL	BDL	BDL	1.73 × 10^1^	2.18 × 10^1^	2.12 × 10^1^	2.27	7.58 × 10^−1^	1.25 × 10^−1^	BDL
Vanillin	BDL	BDL	1.45 × 10^−2^	1.39 × 10^−1^	1.93	4.54	2.25	3.27 × 10^−1^	4.72 × 10^−2^	2.12 × 10^−2^	BDL
Melonal	2.73 × 10^−2^	4.78 × 10^−2^	5.98 × 10^−2^	8.88 × 10^−2^	6.91 × 10^−1^	1.48	7.75 × 10^−1^	1.06 × 10^−1^	5.12 × 10^−2^	2.56 × 10^−2^	1.55 × 10^−2^
3-Hexen-1-ol	1.36 × 10^−1^	1.14 × 10^−1^	1.14 × 10^−1^	8.96 × 10^−2^	9.96 × 10^−2^	6.27 × 10^−2^	9.28 × 10^−2^	9.42 × 10^−2^	7.55 × 10^−2^	7.62 × 10^−2^	7.40 × 10^−2^
Methyl dihydrojasmonate	1.38 × 10^−2^	1.76 × 10^−2^	2.91 × 10^−2^	3.86 × 10^−2^	2.85 × 10^−1^	6.49 × 10^−1^	3.35 × 10^−1^	4.76 × 10^−2^	2.38 × 10^−2^	1.25 × 10^−2^	7.32 × 10^−3^
γ-Decalactone	1.56 × 10^−1^	2.64 × 10^−1^	3.29 × 10^−1^	4.63 × 10^−1^	3.18	7.44	3.62	7.07 × 10^−1^	3.70 × 10^−1^	1.97 × 10^−1^	1.19 × 10^−1^
Chromium	1.74 × 10^1^	1.71 × 10^1^	1.57 × 10^1^	1.75 × 10^1^	1.40 × 10^1^	1.87 × 10^1^	1.68 × 10^1^	1.55 × 10^1^	2.20 × 10^1^	1.53 × 10^1^	1.59 × 10^1^
Nickel	3.60	4.30	3.20	3.30	2.10	1.30 × 10^1^	1.66 × 10^1^	2.37 × 10^1^	1.90 × 10^1^	1.38 × 10^1^	2.67 × 10^1^
Manganese	1.80	1.60	1.40	7.00 × 10^−1^	1.48 × 10^1^	3.70	2.80	2.10	2.30	1.10	2.10
Lead	7.70	7.10	2.10	4.40	4.20	1.90	3.80	3.20	5.70	1.68 × 10^1^	1.80
Aluminum	7.68 × 10^1^	6.61 × 10^1^	4.00 × 10^1^	3.40 × 10^1^	5.93 × 10^1^	1.21 × 10^2^	9.54 × 10^1^	1.73 × 10^2^	1.37 × 10^2^	8.25 × 10^1^	7.78 × 10^1^
Zinc	5.67 × 10^1^	6.55 × 10^1^	5.15 × 10^1^	3.15 × 10^1^	3.12 × 10^1^	1.90 × 10^2^	1.88 × 10^2^	6.62 × 10^1^	1.04 × 10^2^	8.60 × 10^1^	6.44 × 10^1^

*d*: collectible particle diameters (µm); BDL: below detection limit.

**Table 2:** Substances detected in size-segregated ENDS aerosol samples generated by Puff Bar (Grape).

	Mass (ng) Based on 10 Puffs										
MOUDI Stage	1	2	3	4	5	6	7	8	9	10	11
Collectible Size Range (µm)	*d* ≥ 18	18 ≥ *d* ≥ 10	10 ≥ *d* ≥ 5.6	5.6 ≥ *d* ≥ 3.2	3.2 ≥ *d* ≥ 1.8	1.8 ≥ *d* ≥ 1.0	1.0 ≥ *d* ≥ 0.56	0.56 ≥ *d* ≥ 0.32	0.32 ≥ *d* ≥ 0.18	0.18 ≥ *d* ≥ 0.1	0.1 ≥ *d* ≥ 0.0056
Sample Mass	4.15 × 10^3^	2.22 × 10^3^	7.14 × 10^3^	3.08 × 10^4^	3.19 × 10^5^	8.16 × 10^5^	6.56 × 10^5^	1.50 × 10^5^	3.34 × 10^4^	2.06 × 10^3^	8.82 × 10^2^
Nicotine	1.21 × 10^2^	6.40 × 10^1^	2.06 × 10^2^	8.33 × 10^2^	8.53 × 10^3^	1.86 × 10^4^	1.49 × 10^4^	3.34 × 10^3^	1.04 × 10^3^	1.63 × 10^2^	1.81 × 10^2^
Propylene glycol (PG)	BDL	BDL	5.10 × 10^1^	4.59 × 10^2^	1.89 × 10^4^	4.41 × 10^4^	3.63 × 10^4^	9.08 × 10^3^	2.18 × 10^3^	5.94 × 10^1^	3.41 × 10^1^
Glycerol (VG)	1.38 × 10^3^	BDL	3.30 × 10^3^	1.70 × 10^4^	2.87 × 10^5^	7.44 × 10^5^	5.97 × 10^5^	1.34 × 10^5^	2.83 × 10^4^	1.75 × 10^2^	2.94 × 10^1^
Benzoic acid	1.47 × 10^1^	BDL	2.91 × 10^2^	1.10 × 10^2^	1.47 × 10^3^	4.57 × 10^3^	4.12 × 10^3^	1.58 × 10^3^	3.78 × 10^2^	1.72 × 10^1^	1.31
Triethyl citrate	BDL	BDL	1.10	2.36	4.25 × 10^1^	1.47 × 10^2^	1.36 × 10^2^	1.69 × 10^1^	4.97	2.33 × 10^−1^	BDL
Ethyl maltol	7.47 × 10^−1^	5.81 × 10^−1^	9.10 × 10^−1^	4.64	1.07 × 10^2^	2.83 × 10^2^	2.03 × 10^2^	3.73 × 10^1^	9.64	7.27 × 10^−1^	2.86 × 10^−1^
Methyl anthranilate	1.57 × 10^−2^	1.64 × 10^−2^	2.23 × 10^−2^	3.06 × 10^−2^	8.73 × 10^−2^	3.09 × 10^−1^	2.75 × 10^−1^	4.56 × 10^−2^	3.31 × 10^−2^	1.45 × 10^−2^	1.34 × 10^−2^
α-Terpineol	2.98 × 10^−2^	2.29 × 10^−2^	3.45 × 10^−2^	4.18 × 10^−2^	1.14 × 10^−1^	1.89 × 10^−1^	1.38 × 10^−1^	6.80 × 10^−2^	4.18 × 10^−2^	2.58 × 10^−2^	2.24 × 10^−2^
3-Hexen-1-ol	7.58 × 10^−2^	9.03 × 10^−2^	8.99 × 10^−2^	8.18 × 10^−2^	7.84 × 10^−2^	8.64 × 10^−2^	8.36 × 10^−2^	7.91 × 10^−2^	6.90 × 10^−2^	8.50 × 10^−2^	6.76 × 10^−2^
Perillartine	BDL	3.79 × 10^−4^	8.51 × 10^−4^	1.70 × 10^−3^	9.20 × 10^−3^	3.42 × 10^−2^	3.74 × 10^−2^	3.98 × 10^−3^	2.57 × 10^−3^	BDL	BDL
Chromium	9.80	8.60	9.50	8.10	1.24 × 10^1^	7.70	5.10	3.90	4.80	3.80	3.00
Nickel	3.40	1.60	2.00	5.50	2.90	8.30	5.90	2.80	3.50	5.90	1.65 × 10^1^
Manganese	1.20	8.00 × 10^−1^	1.51 × 10^1^	3.80	4.00 × 10^−1^	1.70	1.20	6.00 × 10^−1^	3.00 × 10^−1^	1.00	3.00 × 10^−1^
Lead	1.70	1.30	7.00 × 10^−1^	4.00 × 10^−1^	6.00 × 10^−1^	1.60	5.00 × 10^−1^	3.00 × 10^−1^	5.00 × 10^−1^	1.40	4.00 × 10^−1^
Aluminum	5.39 × 10^1^	5.50 × 10^1^	3.32 × 10^1^	3.89 × 10^1^	3.63 × 10^1^	4.06 × 10^1^	2.66 × 10^1^	2.40 × 10^1^	4.30 × 10^1^	2.56 × 10^1^	2.56 × 10^1^
Zinc	9.17 × 10^1^	3.39 × 10^1^	2.37 × 10^1^	6.43 × 10^1^	6.74 × 10^1^	1.14 × 10^2^	9.58 × 10^1^	2.00 × 10^1^	1.94 × 10^1^	2.70 × 10^1^	1.19 × 10^1^

*d*: collectible particle diameters (µm); BDL: below detection limit.

**Table 3:** Estimated lung-deposited doses and cancer and non-cancer risks of ENDS aerosol generated by Air Bar—Watermelon Ice.

Chemical	Estimated Dose		Cancer Risk Assessment		Non-Cancer Risk Assessment			
	LADD (µg/kg/Day)	ADD (µg/kg/Day)	CSF (µg/kg/Day)^−1^	Cancer Risk	RfD ^a^ (µg/kg/Day)	Non-Cancer Risk (HQ)	Target System	HI
Nicotine	1.80	4.21	-- ^b^	--	-- ^b^	--	--	--
Propylene glycol (PG)	2.69	6.27	-- ^b^	--	-- ^b^	--	--	--
Glycerol (VG)	3.12 × 10^1^	7.27 × 10^1^	-- ^b^	--	-- ^b^	--	--	--
Benzoic acid	1.64	3.82	-- ^b^	--	-- ^b^	--	--	--
Triethyl citrate	2.07 × 10^−3^	4.82 × 10^−3^	-- ^b^	--	-- ^b^	--	--	--
Ethyl maltol	7.91 × 10^−3^	1.85 × 10^−2^	-- ^b^	--	-- ^b^	--	--	--
Benzyl alcohol	9.20 × 10^−4^	2.15 × 10^−3^	-- ^b^	--	-- ^b^	--	--	--
Vanillin	1.40 × 10^−4^	3.20 × 10^−4^	-- ^b^	--	-- ^b^	--	--	--
Melonal	5.00 × 10^−5^	1.10 × 10^−4^	-- ^b^	--	-- ^b^	--	--	--
3-Hexen-1-ol	1.00 × 10^−5^	2.00 × 10^−5^	-- ^b^	--	-- ^b^	--	--	--
Methyl dihydrojasmonate	2.00 × 10^−5^	5.00 × 10^−5^	-- ^b^	--	-- ^b^	--	--	--
γ-Decalactone	2.40 × 10^−4^	5.60 × 10^−4^	-- ^b^	--	-- ^b^	--	--	--
Chromium	1.96 × 10^−3^	4.58 × 10^−3^	5.1 × 10^−1 c^	**1.0 × 10^−3^**	2.9 × 10^−2 d^ (particulate)	0.16	Respiratory	1.14 (Respiratory)
Nickel	1.68 × 10^−3^	3.91 × 10^−3^	9.1 × 10^−4 c^	**1.5 × 10^−6^**	4.0 × 10^−3 e^	0.98	Respiratory	
Manganese	4.60 × 10^−4^	1.07 × 10^−3^	--	--	1.4 × 10^−2 d^	0.08	CNS	0.11 (CNS)
Lead	5.70 × 10^−4^	1.32 × 10^−3^	4.2 × 10^−5 c^	2.4 × 10^−8^	4.3 × 10^−2 f^	0.03	CNS	
Aluminum	1.02 × 10^−2^	2.38 × 10^−2^	-- ^b^	--	-- ^b^	--	--	--
Zinc	1.07 × 10^−2^	2.50 × 10^−2^	-- ^b^	--	-- ^b^	--	--	--

Abbreviations: LADD: lifetime average daily dose; ADD: average daily dose; CSF: cancer slope factor; RfD: reference dose; HQ: hazard quotient; CNS: central nervous system. Bold: exceeds acceptance risk (i.e., cancer risk > 1.0 × 10^−6^; HQ > 1.0; HI > 1.0). ^a^ RfD was calculated as RfC × 20/70 (assuming a inhalation rate of 20 m^3^/day and a default 70 kg body weight). ^b^ No established CSF or RfC. ^c^ Information extracted from CalEPA [32]. ^d^ Based on Reference concentration (RfC) from US EPA [33]. ^e^ Based on RfC from CalEPA [31]. ^f^ Based on RfC from US EPA [34].

**Table 4:** Estimated lung-deposited doses and cancer and non-cancer risks of ENDS aerosol generated by Puff Bar—Grape.

Chemical	Estimated Dose		Cancer Risk Assessment		Non-Cancer Risk Assessment			
	LADD (µg/kg/Day)	ADD (µg/kg/Day)	CSF (µg/kg/Day)^−1^	Cancer Risk	RfD ^a^ (µg/kg/Day)	Non-Cancer Risk (HQ)	Target System	HI
Nicotine	6.73 × 10^−1^	1.57	-- ^b^	--	-- ^b^	--	--	--
Propylene glycol (PG)	1.56	3.63	-- ^b^	--	-- ^b^	--	--	--
Glycerol (VG)	2.54 × 10^1^	5.94 × 10^1^	-- ^b^	--	-- ^b^	--	--	--
Benzoic acid	1.66 × 10^−1^	3.87 × 10^−1^	-- ^b^	--	-- ^b^	--	--	--
Triethyl citrate	4.93 × 10^−3^	1.15 × 10^−2^	-- ^b^	--	-- ^b^	--	--	--
Ethyl maltol	9.23 × 10^−3^	2.15 × 10^−2^	-- ^b^	--	-- ^b^	--	--	--
Methyl anthranilate	1.00 × 10^−5^	3.00 × 10^−5^	-- ^b^	--	-- ^b^	--	--	--
α-Terpineol	1.00 × 10^−5^	2.00 × 10^−5^	-- ^b^	--	-- ^b^	--	--	--
3-Hexen-1-ol	1.00 × 10^−5^	2.00 × 10^−5^	-- ^b^	--	-- ^b^	--	--	--
Perillartine	<1.00 × 10^−5^	<1.00 × 10^−5^	-- ^b^	--	-- ^b^	--	--	--
Chromium	7.60 × 10^−4^	1.78 × 10^−3^	5.1 × 10^−1 c^	**3.9 × 10^−4^**	2.9 × 10^−2 d^ (particulate)	0.06	Respiratory	0.57 (Respiratory)
Nickel	8.70 × 10^−4^	2.04 × 10^−3^	9.1 × 10^−4 c^	8.0 × 10^−7^	4.0 × 10^−3 e^	0.51	Respiratory	
Manganese	2.20 × 10^−4^	5.10 × 10^−4^	--	--	1.4 × 10^−2 d^	0.04	CNS	<0.05 (CNS)
Lead	9.00 × 10^−5^	2.10 × 10^−4^	4.2 × 10^−5 c^	3.8 × 10^−9^	4.3 × 10^−2 f^	<0.01	CNS	
Aluminum	3.87 × 10^−3^	9.03 × 10^−3^	-- ^b^	--	-- ^b^	--	--	--
Zinc	6.13 × 10^−3^	1.43 × 10^−2^	-- ^b^	--	-- ^b^	--	--	--

Abbreviations: LADD: lifetime average daily dose; ADD: average daily dose; CSF: cancer slope factor; RfD: reference dose; HQ: hazard quotient; CNS: central nervous system. Bold: exceeds acceptance risk (i.e., cancer risk > 1.0 × 10^−6^; HQ > 1.0; HI > 1.0). ^a^ RfD was calculated as RfC × 20/70 (assuming an inhalation rate of 20 m^3^/day and a default 70 kg body weight). ^b^ No established CSF or RfC. ^c^ Information extracted from CalEPA [32]. ^d^ Based on Reference concentration (RfC) from US EPA [33]. ^e^ Based on RfC from CalEPA [31]. ^f^ Based on RfC from US EPA [34].

## Data Availability

Some or all data and models that support the findings of this study are available from the corresponding author upon reasonable request.

## Appendix A

**Table A1:** MOUDI 11 stages with collectible particle size range and associated lung deposition fractions.

MOUDI Stage (*i*)	Collectible Particle Diameters (µm)	Average Lung Deposition Fraction (DF)
1	*d* ≥ 18	0.01
2	18 ≥ *d* ≥ 10	0.02
3	10 ≥ *d* ≥ 5.6	0.06
4	5.6 ≥ *d* ≥ 3.2	0.12
5	3.2 ≥ *d* ≥ 1.8	0.17
6	1.8 ≥ *d* ≥ 1.0	0.16
7	1.0 ≥ *d* ≥ 0.56	0.12
8	0.56 ≥ *d* ≥ 0.32	0.08
9	0.32 ≥ *d* ≥ 0.18	0.07
10	0.18 ≥ *d* ≥ 0.1	0.13
11	0.1 ≥ *d* ≥ 0.056	0.25

Data source: this study.
